# New records of *Microgaster deductor* Nixon, 1968 (Hymenoptera: Braconidae: Microgastrinae) for the Holarctic region, with comments on its historical distribution

**DOI:** 10.3897/BDJ.2.e1040

**Published:** 2014-01-03

**Authors:** Jose L Fernandez-Triana

**Affiliations:** † Canadian National Collection of Insects, Ottawa, and Biodiversity Institute of Ontario, University of Guelph, Ottawa, Canada

**Keywords:** Microgastrinae, Nearctic, Palaearctic, Holarctic distribution, COI gene, morphology, species distribution

## Abstract

Four new localities for the species *Microgaster deductor* Nixon (1968) are recorded from the Nearctic (Canada and the United States) and the Palaearctic (Sweden), expanding significantly the range of the species. *Microgaster deductor* seems to be widely distributed in boreal areas of the Holarctic, and it is characterized by unique morphological (tarsal claw structure) and molecular (COI) traits. Preliminary evidence, to be corroborated when more data is available, suggests that the species might have shifted northwards between 1950 and the present day.

## Introduction

*Microgaster deductor* Nixon (1968) is a very distinctive microgastrine wasp (Hymenoptera, Braconidae), and can be easily separated from all Holarctic species of *Microgaster* based on its tarsal claws, which have a lobe (Nixon 1968) (Fig. 2). Until very recently the species was thought to be restricted to northern areas of Europe, in the Western Palaearctic (Huflejt 1997, Koponen and Vikberg 1984). However, a few years ago the species was recorded from Churchill, Manitoba, Canada (Fernandez-Triana et al. 2011, Fernández-Triana 2010). That locality was the first record of the species for the Nearctic, and suggested that *Microgaster deductor* could be more widely distributed in sub-Arctic or Arctic areas of the Holarctic (Europe and North America).

Here *Microgaster deductor* is recorded from four additional localities, three in the Nearctic and one in the Palaearctic, which expand significantly the known range of the species. Morphological and molecular data that serve as diagnostic tools are presented, and the potential of a shift northwards of the species distribution is briefly discussed.

## Materials and methods

All the specimens studied for this paper are deposited in the Canadian National Collection of Insects (CNC), Ottawa, Canada. The only exception is one female deposited in the Biodiversity Institute of Ontario, which was not examined directly. Instead it was studied based on a single photo (habitus, lateral) and associated DNA barcode, both freely available as public data in the Barcode of Life Data Systems (BOLD) (http://www.boldsystems.org/).

Additionally, 40 specimens of *Microgaster* spp., representing contemporary collecting from the Swedish Malaise Trap Project (http://www.artdata.slu.se/svenskaartprojektet/malaisetrap.asp) were examined, although no specimen of *Microgaster deductor* was found in those samples.

The historical data on the distribution of the species was extracted from the original references (Huflejt 1997, Koponen and Vikberg 1984, Nixon 1968) and compared against the new records reported here. A map with the distribution of the species was generated using SimpleMappr (Shorthouse 2010).

## Taxon treatments

### Microgaster deductor

Nixon, 1968

#### Materials

- **Type status:** Holotype. **Occurrence:** individualCount: 1; sex: female; **Location:** country: Finland; stateProvince: Lapland; verbatimLocality: Ivalo; **Record Level:** institutionCode: BMNH
- **Type status:** Paratype. **Occurrence:** individualCount: 2; sex: 1 female, 1 male; **Location:** country: Finland; stateProvince: Lapland; verbatimLocality: Ivalo; **Record Level:** institutionCode: BMNH
- **Type status:** Paratype. **Occurrence:** individualCount: 1; sex: female; **Location:** country: Sweden; stateProvince: Lapland; verbatimLocality: Torneträsk; **Record Level:** institutionCode: BMNH
- **Type status:** Other material. **Occurrence:** recordedBy: Jose Fernández-Triana; individualCount: 3; sex: 1 female, 2 males; **Location:** country: United States; stateProvince: Alaska; verbatimLocality: Unalakleet; verbatimLatitude: 63.878889; verbatimLongitude: -160.789722; **Event:** eventDate: 27 Jun 1961, 28 Jun 1961, 4 Jul 1961; **Record Level:** institutionCode: CNC
- **Type status:** Other material. **Occurrence:** recordedBy: Jose Fernández-Triana; individualCount: 4; sex: females; **Location:** country: Sweden; stateProvince: Lapland; verbatimLocality: Abisko; verbatimLatitude: 68.35; verbatimLongitude: 18.816667; **Event:** eventDate: 29 Jul 1951, 9 Aug 1951, 15 Aug 1951; **Record Level:** institutionCode: CNC
- **Type status:** Other material. **Occurrence:** recordedBy: Jose Fernández-Triana; individualCount: 1; sex: female; **Location:** country: Sweden; stateProvince: Lapland; verbatimLocality: Abisko; verbatimElevation: 400 m; verbatimLatitude: 68.35; verbatimLongitude: 18.816667; **Event:** eventDate: 31 Jul 1960; **Record Level:** institutionCode: CNC
- **Type status:** Other material. **Occurrence:** recordedBy: Jose Fernández-Triana; individualCount: 1; sex: female; **Location:** country: Canada; stateProvince: Northwest Territories; verbatimLocality: Tuktoyaktuk; verbatimLatitude: 66.4445; verbatimLongitude: -133.032; **Event:** samplingProtocol: Sweeping; eventDate: 14 Jul 2010; **Record Level:** institutionCode: CNC
- **Type status:** Other material. **Occurrence:** recordedBy: Jose Fernández-Triana; individualCount: 1; sex: female; **Location:** country: Canada; stateProvince: Yukon Territory; verbatimLocality: Herschel Island; verbatimLatitude: 69.571; verbatimLongitude: -138.902; **Event:** eventDate: 29 Jul 2008; **Record Level:** institutionCode: BIO
- **Type status:** Other material. **Occurrence:** recordedBy: Jose Fernández-Triana; individualCount: 35; **Location:** country: Canada; stateProvince: Manitoba; verbatimLocality: 23 km E of Churchill; verbatimLatitude: 58.734; verbatimLongitude: -93.82; **Event:** eventDate: 12 Jul 1952, 18 Jul 1952, 23 Jul 1952, 28 Jul 1952, 29 Jul 1952, 3 Aug 1952, 5 Aug 1952; **Record Level:** institutionCode: CNC
- **Type status:** Other material. **Occurrence:** recordedBy: Jose Fernández-Triana; individualCount: 6; **Location:** country: Canada; stateProvince: Manitoba; verbatimLocality: Warkworth Creek nr. Churchill; verbatimLatitude: 58.375; verbatimLongitude: -93.875; **Event:** eventDate: 29 Jun 1952, 7 Jul 1952, 3 Aug 1952; **Record Level:** institutionCode: CNC

## Discussion

New distribution records for *Microgaster deductor* in the Nearcitc include three localities: Unalakleet, Alaska (United States), and Tuktoyaktuk, Northwest Territories and Herschel Island, Yukon Territory (Canada). They expand considerably the distribution of the species in the Holarctic, and the Alaskan record suggests the possibility that the species might also be in Siberia, Russia (although that could not be confirmed during this study). The new record in the Palaearctic is from Abisko in Sweden, a locality very close to that of Torneträsk, where one of the paratypes, included by Nixon (Nixon 1968) in his original description of the species, was collected. It should be clarified that Nixon misspelled the name of that locality as Tornekrask.

**Figure 1.**
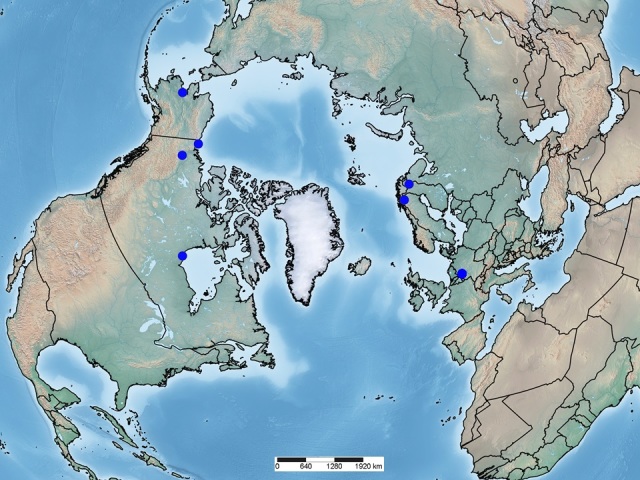
Distribution of *Microgaster deductor*.

Considering all the available information for *Microgaster deductor*, the species is relatively widely distributed in Arctic or sub-Artic localities of the Holarctic region, mostly from 59°–70°N (Fig. 1), and in areas covered by boreal forest, or at the interface of boreal forest with tundra vegetation. The only exception is one female specimen from Poland (Radziejowice near Mszczonów, at 52°N), determined in the 1980's by P. Marczak as belonging to the species (Huflejt 1997). This record might be based on a misidentification of the species, but without examining the specimen it is impossible to re-asses its status.

**Figure 2.**
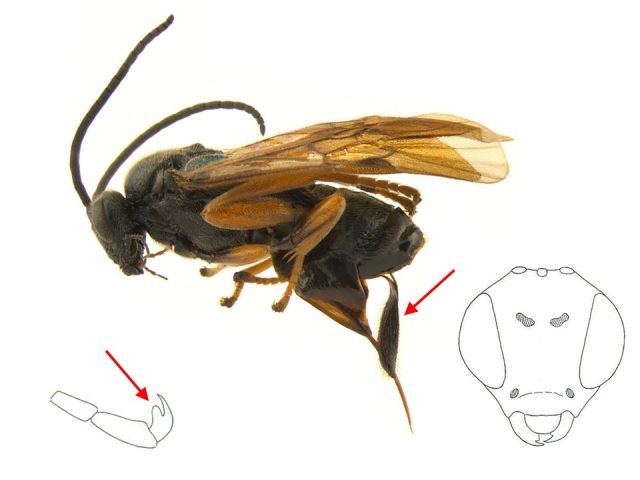
*Microgaster deductor* specimen from Churchill, Manitoba. Red arrows show a lobed tarsal claw and the ovipositor sheaths. Frontal view of head modified from Nixon (1968).

**Figure 3.**
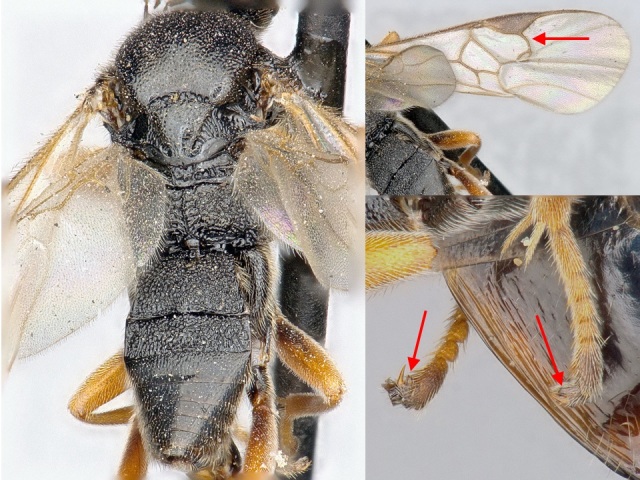
*Microgaster deductor* specimen from Abisko, Sweden. Habitus, dorsal view (left), details of fore wing (top right) and tarsal claws (bottom right). Red arrows show tarsal claw lobes, and vein r of fore wing.

Morphologically, this species is rather uniform (Figs 2, 3), without any significant external difference between the Palaearctic and Nearctic specimens examined. There is some variation in the pterostigma, which has a pale spot anteriorly in some specimens (e.g., http://www.boldsystems.org/index.php/Taxbrowser_Taxonpage?taxon=Microgaster+deductor&searchMenu=taxonomy&query=Microgaster+deductor) but in others the pale spot is reduced (e.g., Fig. 3). Since its original description, *Microgaster deductor* has been considered as highly aberrant on account of its lobed tarsal claws (Nixon 1968) (Figs 2, 3). No other described species of *Microgaster* in the Holarctic has this feature. Other diagnostic features are the relatively elongated face, fore wing with vein r strongly inclined towards outer margin of wing, and the shape of the ovipositor sheaths (Figs 2, 3).

The two specimens recently collected (2008 and 2010) rendered full DNA barcodes – a section of 658 base pairs of the mitochondrial COI gene. Among the material collected in Churchill in 1952, one specimen rendered about half a barcode (320 base pairs), and for another 33 specimens short sequences of 129-144 base pairs were obtained. All sequences were identical, with the shorter ones perfectly matching the corresponding section of the full barcode obtained from the two recent specimens. The closest sequences in BOLD (which contains a library of over 2.7 million sequences, including more than 800 specimens and 70 species of *Microgaster*) differed from *Microgaster deductor* by 46 base pairs (7%). Full data of the sequences and specimens can be freely accessed in BOLD from the public projects with codes CNCAS, HARC and WOMIA. The nucleotide sequence of the full barcode of *Microgaster deductor*, in FASTA format is:

AATATTATATTTTTTATTTGGATTATGATCTGGGATATTAGGATTTTCAATAAGATTAAT TATTCGGTTAGAATTAGGTATTCCTGGTAGATTAATTGGAAATGACCAAATTTATAATAG AATTGTGACATCTCATGCTTTTATTATAATTTTTTTTATAGTAATACCTGTAATAATTGG GGGATTTGGAAATTGATTAATTCCTTTAATATTAGGTTCTCCAGATATATCATTTCCACG TATAAATAATATAAGATTTTGATTATTAATTCCATCATTAATATTATTAATTTCTAGGAT ATTTATTAATGTGGGTGTTGGAACTGGATGAACAGTTTATCCTCCATTATCATTAATTTT AGGTCATGGAGGTATATCTGTCGATTTAGGAATTTTTTCATTACATTTAGCTGGAGCTTC TTCAATTATAGGTGCAGTAAATTTTATTACAACAATTATAAATATACGAGTTAAAATATA TTTAATAGATAAAATATCTTTATTTTCTTGATCAGTTTTTATTACTGCAATTTTATTATT AATATCTTTACCTGTTTTAGCAGGTGCTATTACAATATTATTAACTGATCGTAATATTAA TACTAGATTTTTTGATCCTGCTGGTGGAGGGGATCCTATTTTATATCAACATTTATTT

Based on the studied molecular and morphological data, *Microgaster deductor* is a very distinctive and defined species across its whole Holarctic range. At present nothing is known of the Lepidoptera host(s) species that this wasp parasitizes.

The relatively scarce information about historical (1950–1960) and present (2008–2010) distribution of *Microgaster deductor*, seems to suggest that its range might have shifted northwards recently. For example, the species was the most commonly collected Microgastrinae in Churchill during a period of intense study of insects in the area around the 1950's. However, it has never been found there again, in spite of even more intense collecting efforts carried out in the same locality between 2006–2011 (Fernandez-Triana et al. 2011). Similarly, the species was collected in Alaska, Finland and Sweden between 1950 and 1960, but has not been found later there, in spite of extensive collecting being done in those areas for the past three decades [e.g. Koponen collected 370 specimens of Braconidae in or near the holotype locality in 1983, but did not find *Microgaster deductor* (Koponen and Vikberg 1984); also, based on the material I have examined so far from the Swedish Malaise Trap Project (including hundreds of Microgastrinae specimens), this project has not recovered the species either]. Conversely, the only two recent records of *Microgaster deductor* correspond to Canadian localities at 69–70°N, which had similarly been sampled in the 1940–1960 without finding any record of the species. These two localities are 6–10° north of the Canadian collections from the 1950–1960.

However, both historical and current day distribution patterns are likely to be biased by incomplete sampling efforts. The study of more specimens and more localities will be necessary before reaching any conclusions on this topic.

## Supplementary Material

XML Treatment for Microgaster deductor
